# Supplementary material for: Diabetic macular edema: Safe and effective treatment with intravitreal triamcinolone acetonide (Taioftal)
Source: PLoS One. 2021 Oct 1;16(10):e0257695. doi: 10.1371/journal.pone.0257695 (PMC8486126; doi:10.1371/journal.pone.0257695)
Supplement: Attachment — Submitted filename: Response to Reviewers.docx [file pone.0257695.s001.docx]

To Reviewers

First of all, I thank you all for the time dedicating to evaluate this manuscript giving your personal scientifically considerations. It has been a pleasure to revise it, according to your careful observations and useful suggestions.

To Reviewer 1, you were perfectly right to underline the fact I would have stressed about the difference between triamcinolone with preservatives and preservative-free formulation. I focused on a specific preservative-free molecule (Taioftal). According with papers you pointed out, I enriched this manuscript with considerations about the difference between treatments with preservative or preservative-free triamcinolone. I totally agree about the next chance to make comparative studies between different types of triamcinolone.

To Reviewer 2, I rewrote the abstract adding some important data as you requested. I collected all data during my routinely outpatient clinics, so there was just me as researcher. However, the statistical analysis was performed with the essential help of Professor Claudio Bonifazzi from the University of Ferrara. Also, the design of the study is due to Professor Francesco Parmeggiani from the University of Ferrara. I really apologize for my shameful absent-mindedness about the list of Authors. About Figures and Tables, I think there are not busy but rich of information and data. With a deeper look at graphs and legends, you can understand the sense of the entire paper. Anyway, I tried to explain figures as much as I can because I recognize their complexity. Thank you for the interesting paper you reported about the rebound phenomenon after IVTA. Authors pointed out this phenomenon from baseline at 2 months after IVTA injection. Personally, I have never experienced it when treating diabetic macular edema, but rarely when treating macular edema post-occlusion.

Thank you all again for your help to improve this manuscript.
